# Antibody responses to *Schistosoma mansoni* schistosomula antigens

**DOI:** 10.1111/pim.12591

**Published:** 2018-10-14

**Authors:** Moses Egesa, Lawrence Lubyayi, Frances M. Jones, Angela van Diepen, Iain W. Chalmers, Edridah M. Tukahebwa, Bernard S. Bagaya, Cornelis H. Hokke, Karl F. Hoffmann, David W. Dunne, Alison M. Elliott, Maria Yazdanbakhsh, Shona Wilson, Stephen Cose

**Affiliations:** ^1^ Department of Medical Microbiology School of Biomedical Sciences Makerere University College of Health Sciences Kampala Uganda; ^2^ Medical Research Council/Uganda Virus Research Institute and London School of Hygiene & Tropical Medicine Uganda Research Unit Entebbe Uganda; ^3^ Department of Pathology University of Cambridge Cambridge UK; ^4^ Department of Parasitology Leiden University Medical Center Leiden The Netherlands; ^5^ Institute of Biological, Environmental & Rural Sciences Aberystwyth University Aberystwyth UK; ^6^ Vector Control Division Ministry of Health Kampala Uganda; ^7^ Department of Immunology and Molecular Biology School of Biomedical Sciences Makerere University College of Health Sciences Kampala Uganda; ^8^ Department of Clinical Research London School of Hygiene & Tropical Medicine London UK

**Keywords:** antibody responder, IgE, reinfection, *Schistosoma mansoni*, schistosomula antigens

## Abstract

While antigens from *Schistosoma* schistosomula have been suggested as potential vaccine candidates, the association between antibody responses with schistosomula antigens and infection intensity at reinfection is not well known. *Schistosoma mansoni*‐infected individuals were recruited from a schistosomiasis endemic area in Uganda (n = 372), treated with 40 mg/kg praziquantel (PZQ) and followed up at five weeks and at one year post‐treatment. Pre‐treatment and five weeks post‐treatment immunoglobulin (Ig) E, IgG1 and IgG4 levels against recombinant schistosomula antigens rSmKK7, rSmLy6A, rSmLy6B and rSmTSP7 were measured using ELISA. Factors associated with detectable pre‐treatment or post‐treatment antibody response against the schistosomula antigens and the association between five‐week antibody responses and one year post‐treatment reinfection intensity among antibody responders were examined. Being male was associated with higher pre‐treatment IgG1 to rSmKK7, rSmLy6a and AWA. Five weeks post‐treatment antibody responses against schistosomula antigens were not associated with one year post‐treatment reinfection intensity among antibody responders’ antibody levels against rSmKK7, rSmLy6B and rSmTSP7 dropped, but increased against rSmLy6A, AWA and SEA at five weeks post‐treatment among antibody responders. *S. mansoni*‐infected individuals exhibit detectable antibody responses to schistosomula antigens that are affected by treatment. These findings indicate that schistosomula antigens induce highly varied antibody responses and could have implications for vaccine development.

## 1 INTRODUCTION

Approximately 206 million people worldwide required treatment for schistosomiasis in 2016.^1^ Control programmes in affected countries have reduced the morbidity associated with schistosomiasis,^2^ yet despite this, treatment is unable to prevent reinfection with *Schistosoma* species, and consequently, transmission of schistosomiasis remains a global problem. Mass drug administration with praziquantel (PZQ) alone may not ultimately control schistosomiasis,^3^ and therefore, a solution such as a prophylactic vaccine is needed to provide long‐term immunity and preferably complement the PZQ‐based schistosomiasis control strategy.^4^ There is currently no vaccine against schistosomes, and only a few vaccine candidates are in clinical trials. Despite the limited success, defined antigens continue to be investigated as vaccine targets. Among these are those from the larvae of the schistosome, known as schistosomula. The schistosomula develop when free‐living cercariae penetrate host skin, lose their bifurcated tails and shed their cercarial glycocalyx coat in the skin in a process called transformation. Early work on schistosome parasite biology showed that transformation makes the skin schistosomula vulnerable to killing by host immune responses.^5^ This killing is mediated through complement fixation 6, 7 and antibody (IgE, IgA and IgG)‐dependent cell‐mediated cytotoxicity (ADCC).^8^, ^9^, ^10^ The antibodies coat the schistosomula and allow eosinophils to kill the parasite in vitro.^11^, ^12^ This suggests that the schistosomula are a potential source of vaccine candidates.

By examining the *S. mansoni* transcriptome, genes that are highly expressed or upregulated during the schistosomula stage compared to the infective cercariae can be identified.^13^ Indeed, DNA microarray‐based analysis of the *S. mansoni* life cycle has revealed schistosomula‐enriched gene products, such as those coding for SmKK7 (smp_194830), *S. mansoni* lymphocyte antigen 6 isoforms A and B (SmLy6A; smp_019350, SmLy6B; smp_105220) and *S. mansoni* tetraspanin 7 (SmTSP7; smp_099770).^14^ SmLy6A and SmLy6B are probably glycophosphatidylinositol (GPI)‐anchored antigens, found in schistosomula,^13^, ^15^ but also in adult tegumental and mesenchymal tissues.^16^, ^17^ Although SmLy6A and SmLy6B are homologues of human CD59, which inhibits the formation of the complement membrane attack complex, they do not inhibit complement fixation and their function remains unknown.^17^ SmKK7 is a putative immunomodulatory protein found in the peripheral nervous system of *S. mansoni* adults 18 and upregulated in 7‐ and 14‐day schistosomula.^15^ SmTSP7 is a membrane‐spanning tetraspanin with unknown function but has been identified in the tegument of the adult worm.^19^

Epidemiological studies have shown that specific host antibodies to *Schistosoma* antigens contribute to immunity to schistosomiasis. For instance, antibody responses targeting *S. mansoni* adult worm antigens are associated with resistance or susceptibility to reinfection of people at risk in endemic areas.^20^, ^21^ Although adult worm antigen‐specific IgE is associated with resistance against reinfection, it is important to note that vaccine candidates that induce IgE production may not be safe for use, particularly in previously helminth‐infected individuals. This was accentuated during a trial involving a hookworm vaccine candidate that was discontinued when previously hookworm‐infected participants developed an IgE‐mediated allergic reaction.^22^, ^23^ The same hookworm vaccine had earlier been shown to be safe with healthy hookworm‐naive individuals.^24^ This suggests that helminth (including *Schistosoma*) antigens should be pre‐clinically screened using sera from infected people to determine whether antigen‐specific IgE is present.

On the other hand, IgG4 blocks IgE‐mediated protective immunity by competing with IgE to bind shared epitopes 25 and engaging the inhibitory IgG receptor, FcγIIb, that downregulates signalling from the IgE receptor, FcεRI, on effector cells.^26^, ^27^ As a result, activation of effector cells is inhibited 28 and protective responses may be less effective. Indeed, *Schistosoma*‐infected people, especially children (who are the most susceptible to reinfection), produce high levels of IgG4.^29^, ^30^ Although antibody responses (IgE and IgG) to crude purified extracts of schistosomula have been shown to contribute to human resistance,^31^, ^32^ few studies have looked at antibody responses to recombinant schistosomula antigens.^33^

The aim of this study was to determine how infection intensity, age and sex affect antibody responses to the recombinant *S. mansoni* schistosomula antigens rSmLy6A, rSmLy6B, rSmKK7 and rSmTSP7 in an endemic population. We also analysed the effect of treatment on the antibody responses against these recombinant antigens and the correlation between the antibody and cytokine responses (in the companion paper). Finally, we examined whether pre‐ or five weeks post‐treatment antibody responses were associated with reinfection one year later.

## 2 MATERIALS AND METHODS

### 2.1 Ethical statement

Informed consent was obtained from adults in Namoni to participate in this study. Children gave written assent to participate in the study. The Makerere University School of Biomedical Sciences Higher Degrees Research and Ethics Committee (reference number SBS 300) and the Uganda National Council for Science and Technology (reference number HS 1040) approved this work.

### 2.2 Recruitment of study participants

**Figure 1:**
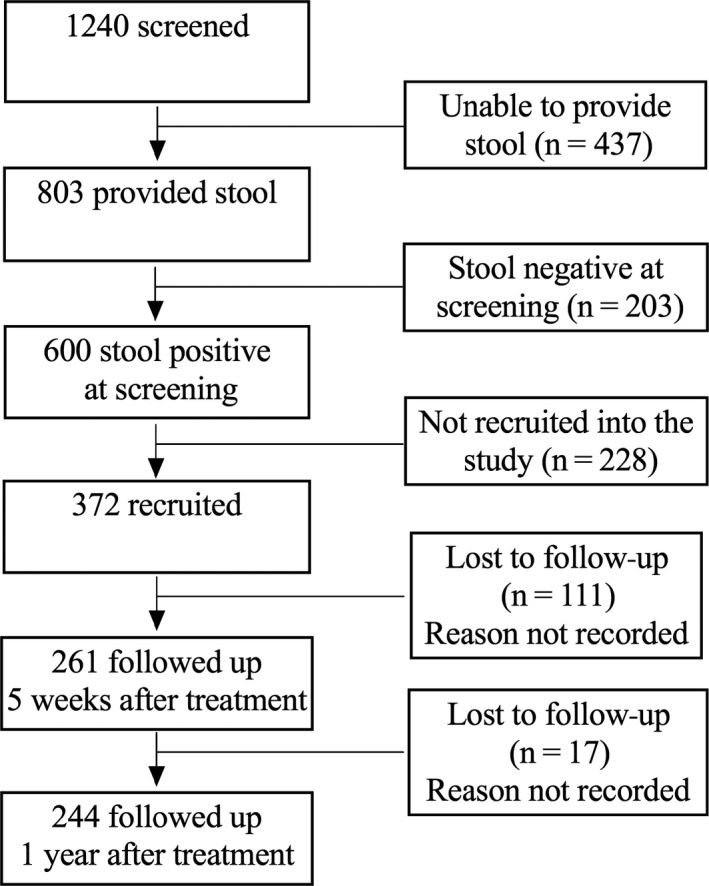
The study profile describing the screening, recruitment and follow‐up of the study participants

Individuals were recruited from Namoni village, a Ugandan fishing community that is endemic with schistosomiasis at the shore of Lake Victoria as part of TheSchistoVac study (http://www.theschistovac.eu) to develop antigens for a prophylactic schistosomiasis vaccine. A total of 372 individuals aged between 6 and 40 years were recruited from Namoni (Figure 1). TheSchistoVac cohort in Namoni has been described elsewhere.^34^ In September 2011, the participants were treated with two doses of praziquantel (40 mg/kg) one week apart and followed up for 5 weeks and a year. Infection intensity was determined from stool samples collected pre‐treatment, at 5 weeks for efficacy of the PZQ treatment and one year for reinfection. Participants were asked to donate a venous blood sample immediately before the first praziquantel treatment and a further blood sample five weeks later. Plasma samples were separated, stored and later tested for antibody responses.

Of those recruited and followed up to the end of the study, 244 (65.6%) completed the study. However, 240 (64.5%) of those recruited had complete data on infection intensity, age, sex and levels of IgG1, IgG4 and IgE to AWA, SEA and the antigens at baseline, five weeks and one year after PZQ treatment (Figure 1).

The water contact behaviour of the study participants was obtained and recorded before treatment and included the duration of contact (time spent in the lake) and whether or not the participants bathed, swam, played, fished or processed fish, washed utensils or clothes in the lake, or engaged in transport across the lake or engaged in farm irrigation using lake water. The water contact behaviour was self‐reported during questionnaire interviews.

### 2.3 Stool examination by microscopy

Before treatment, three stool samples were collected on three consecutive days in the morning and two thick smears made from each sample. The six slides were examined using the Kato Katz method for the number of *S. mansoni* eggs as previously described.^35^ The egg count was the average of the six slides from the three samples. The infection intensity was calculated by multiplying the average egg count with 24 and expressed as egg count per gram of stool. The infection intensity of the participants was classified as either light (1‐99 epg), moderate (100‐399 epg) or heavy (>400 epg) based on the WHO criteria.^36^ Additional stool samples were collected five weeks and one year after PZQ treatment. Of the 240 infected individuals, 186 (77.5%) had no eggs (putatively cured)^37^ by the Kato Katz method performed on three stool samples five weeks after PZQ treatment. One year after PZQ treatment, 154 (82.8%) of those cured were egg positive, determined by the Kato Katz method performed on six smears from three stool samples. Reinfection was defined as the presence of eggs at one year after PZQ treatment.^38^

### 2.4 *Schistosoma mansoni* antigens

The antigens used in this study were *S. mansoni* adult worm (AWA) and egg antigens (SEA) and recombinant schistosomula antigens rSmKK7, rSmLy6A, rSmLy6B and rSmTSP7. These antigens were identified as highly expressed products in the schistosomula life cycle stage after screening the *Schistosoma* transcriptome using DNA microarrays, as previously described.^14^, ^16^ Specifically, a >5‐fold increase in expression when comparing normalized expression averages from snail (egg, miracidia, mother sporocyst and daughter sporocyst) to schistosomula (3‐h, 24‐h, 3‐day and 6‐day schistosomula) life stages was observed.^16^ Recombinant antigens were expressed in *Escherichia coli* and purified using methods as previously described for rSmKK7 (smp_194830),^14^ rSmLy6A and rSmLy6B.^15^ For SmTSP7, 78 amino acids representing the extracellular loop 2 of the protein (108‐185AA; defined by TMHMM2.0 software 39 were expressed using the same vector, expression parameters and purification methods as SmLy6A and SmLy6B. The selected proteins used in this study were screened for in silico similarity to known allergens, and any with a similarity were discarded from the pipeline. The selected proteins were further screened for IgE reactivity using sera from a schistosomiasis endemic cohort, Musoli. It was found that Musoli residents had minimal IgE reactivity to these proteins, and the antigens were therefore selected for further study.

### 2.5 Blood collection and estimation of antibody responses

Plasma samples were separated from whole blood before and five weeks after PZQ treatment by centrifugation and stored at ‐20°C until ready for use. The concentrations of IgG1, IgG4 and IgE specific for the schistosomula antigens (rSmKK7, rSLy6A, rSmLy6B, rSmTSP7) and the crude parasite antigens (AWA and SEA) were quantified using ELISA as previously described.^40^ Briefly, the well surfaces of 384‐well Microlon 600 high‐binding plates (Greiner, Meadville, PA, USA) were washed with distilled water and coated overnight at 4°C with 15 μL/well of antigen diluted in sodium bicarbonate solution. The saturating concentrations of rSmKK7, rSmLy6A, rSmLy6B, rSmTSP7, AWA and SEA used were 13.40, 6.25, 9.60, 6.25, 8 and 1.25 μg/mL, respectively. The plates were blocked for 1 h, and sera from the infected Ugandan individuals and uninfected nonendemic European control samples were diluted 1/200 for IgG1 and IgG4 and 1/20 dilution for IgE, plated and incubated overnight at 4°C. The serum samples were tested in duplicate. Diluted monoclonal mouse anti‐human IgG1, IgG4 and IgE biotin (Pharmingen, San Diego, CA, USA) were used and incubated at room temperature for 2 h for IgG1 and IgG4 or +4°C overnight for IgE, respectively. Detection was performed using Poly‐HRP streptavidin complex (Mast Group, UK) and OPD substrate solution (Sigma, Ronkonkoma, NY, USA). The reaction was stopped using 2M sulphuric acid and absorbance read at test wavelength 490 nm and reference wavelength 630 nm on a PowerWave HT microplate reader (BioTek Instruments Inc., Winooski, VT, USA). Gen 5 software (BioTek Instruments Inc.) generated antibody responses from the standard curve.

### 2.6 Cytokine responses to schistosomula antigens

Stored peripheral blood mononuclear cells were thawed, stimulated with rSmKK7, rSLy6A, rSmLy6B and rSmTSP7 and the supernatant collected after 24 h and tested for cytokines, chemokines and growth factors as described in the companion paper (Egesa unpublished).

### 2.7 Statistical methods

Antibody (IgG1, IgG4 and IgE) levels against the schistosomula antigens before treatment were categorized as antibody responders and nonresponders. Antibody responders were participants with antibody levels greater than the mean plus 3 standard deviations against the schistosomula antigens detected in the plasma of 26 nonendemic European individuals. Factors associated with detectable pre‐treatment or post‐treatment antibody (IgG1, IgG4 and IgE) responses against crude schistosome and schistosomula antigens were determined using multiple logistic regression. The correlation between the antibody levels and cytokine responses to schistosomula antigens among antibody responders was investigated using Spearman's rank correlation. Multivariable logistic regression was used to investigate whether detectable five weeks post‐treatment antibody responses were associated with one year post‐treatment reinfection intensity. Because antibody levels were not normally distributed, the levels were log‐transformed and the transformed antibody levels compared before and 5 weeks after treatment among responders, using the paired *t*‐test. Statistical analysis was performed using Stata version 13 (Stata Corp, College Station, TX, USA) and graphs drawn using GraphPad Prism version 6.0 g (GraphPad Software, Inc., , San Diego, CA, USA).

## 3 RESULTS

### 3.1 Demographics of the study participants

Table 1 shows the demographics of the Namoni study participants at baseline. The age range of the study participants was 6‐40 years. There were slightly more females than males. The study participants mainly had a heavy infection intensity (>400 epg) before treatment.

**Table 1:** Baseline demographics of the study participants (N = 240)

Factor	Level	n (%)
Sex	Female	135 (56)
Age (years)	6‐9	74 (31)
	10‐13	76 (32)
	>14	90 (37)
Pre‐treatment infection intensity (epg)	Light (1‐99)	44 (18)
	Moderate (100‐399)	41 (17)
	Heavy (>400)	155 (65)

The majority of the study participants spent no longer than one hour a day in the lake (Table S1). However, the male study participants spent more time in the lake than their female counterparts (*X* ^2^ (5, N = 226) = 20.24, *P* = 0.001). There was no age difference in water contact behaviour (*X* ^2^ (10, N = 226) = 16.37, *P* = 0.089).

### 3.2 Number of antibody responders to the schistosomula antigens

The number and percentage of responders are shown in Table 2. There was a substantial number of IgG1 responders to the schistosomula antigens rSmKK7 (63.3%), rSmLy6b (76.3%) and rSmTSP7 (43.8). Generally, the prevalence of IgG4 responders to the schistosomula antigens was below 22%. IgE responders to rSmKK7 (26.3%) and SmLy6b (43.3%) were observed among the study participants. The number of IgG1, IgG4 and IgE responders to the crude *S. mansoni* AWA and SEA was relatively high compared to the schistosomula antigens.

**Table 2:** Antibody responders to the schistosomula antigens

Antibody	Antibody responders, n (%)^a^					
	SmKK7	SmLy6a	SmLy6b	SmTSP7	AWA	SEA
IgG1	152 (63.3)	32 (13.33)	182 (76.3)	105 (43.8)	220 (91.7)	223 (92.9)
IgG4	51 (21.3)	9 (3.8)	28 (11.7)	15 (6.3)	219 (91.3)	233 (97.1)
IgE	63 (26.3)	9 (3.8)	104 (43.3)	13 (5.4)	123 (51.3)	75 (31.3)

a As a percentage of the 240 individuals whose data were analysed.

### 3.3 Age and sex were associated with pre‐treatment and post‐treatment antibody responses against the crude antigens among antibody responders

Tables 3 and S2 show factors associated with pre‐treatment and five weeks post‐treatment antibody responses against crude *S. mansoni* antigens, respectively.

**Table 3:** Factors associated with pre‐treatment IgG1, IgG4 and IgE detectable response against crude *Schistosoma mansoni* antigens

Antigen	Factor	Level	IgG1		IgG4		IgE	
			Adjusted^a^ OR (95% CI)	*P*‐value	Adjusted^a^ OR (95% CI)	*P*‐value	Adjusted^a^ OR (95% CI)	*P*‐value
AWA	Sex	Female	1	0.013	1	0.125	1	0.005
		Male	5.00 (1.40‐17.86)		2.20 (0.80‐5.99)		2.15 (1.25‐3.68)	
	Age (years)	6 to 9	1	0.089	1	0.203	1	0.031
		10 to 13	4.09 (0.78‐21.37)		3.32 (0.84‐13.24)		2.19 (1.12‐4.29)	
		14+	0.73 (0.25‐2.10)		0.54 (0.15‐2.01)		2.17 (1.13‐4.14)	
SEA	Sex	Female	1	0.290	1	0.368	1	0.029
		Male	1.81 (0.60‐5.48)		2.17 (0.40‐11.73)		1.87 (1.07‐3.26)	
	Age (years)	6 to 9	1	0.037	1	0.560	1	0.854
		10 to 13	4.67 (0.51‐43.19)		3.54 (0.35‐35.33)		0.82 (0.41‐1.65)	
		14+	0.41 (0.13‐1.35)		1.41 (0.26‐7.37)		0.88 (0.45‐3.00)	

OR, odds ratio.

a Adjusted for either sex or age.

Before treatment, being male was associated with higher pre‐treatment IgG1 to AWA (*P* = 0.013) and IgE to AWA (*P* = 0.005) and SEA (*P* = 0.029) after adjusting for gender. Being 10 years and over was associated with higher IgE levels against AWA (*P* = 0.031).

Table S2 shows factors that were associated with five weeks post‐treatment antibody responses against the crude *S. mansoni* antigens, respectively. Five weeks following treatment, being male was associated with higher IgE to AWA (*P* = 0.015) after adjusting for age. Being 10 years and over was associated with higher five weeks post‐treatment IgE levels against AWA after adjusting for gender (*P* = 0.025).

### 3.4 Being male was associated with pre‐treatment and post‐treatment antibody responses against the recombinant schistosomula antigens among antibody responders

Tables 4 and S3 show factors that were associated with pre‐treatment and five weeks post‐treatment antibody responses against the recombinant schistosomula antigens among responders, respectively. Before treatment, being male was associated with higher IgG1 to rSmKK7 (*P* = 0.009) and rSmLy6a (*P* = 0.006). Five weeks following treatment, being male was associated with higher IgG4 levels against SmKK7 (*P* = 0.0001) and SmLy6B (*P* = 0.045).

**Table 4:** Factors associated with pre‐treatment IgG1, IgG4 and IgE detectable responses against *Schistosoma mansoni* schistosomula antigens

Antigen	Factor	Level	IgG1		IgG4		IgE	
			Adjusted^a^ OR (95% CI)	*P*‐value	Adjusted^a^ OR (95% CI)	*P*‐value	Adjusted^a^ OR (95% CI)	*P*‐value
SmKK7	Sex	Female	1	0.009	1	0.143	1	0.625
		Male	2.09 (1.20‐3.65)		1.62 (0.85‐3.07)		0.86 (0.48‐1.56)	
	Age (years)	6 to 9	1	0.739	1	0.019	1	0.943
		10 to 13	1.31 (0.66‐2.60)		1.96 (0.92‐4.18)		0.88 (0.42‐1.84)	
		14+	1.15 (0.60‐2.21)		0.66 (0.28‐1.53)		0.96 (0.60‐2.21)	
SmLy6a	Sex	Female	1	0.006	1	0.073	1	0.549
		Male	3.13 (1.39‐7.02)		4.37 (0.87‐21.89)		1.5 (0.39‐5.91)	
	Age (years)	6 to 9	1	0.441	1	0.380	1	0.716
		10 to 13	0.93 (0.37‐2.28)		1.18 (0.27‐5.04)		0.51 (0.09‐2.85)	
		14+	1.15 (0.21‐1.45)		0.25 (0.03‐2.31)		0.65 (0.13‐3.08)	
SmLy6b	Sex	Female	1	0.304	1	0.713	1	0.438
		Male	1.39 (0.74‐2.59)		1.17 (0.51‐2.67)		0.81 (0.48‐1.38)	
	Age (years)	6 to 9	1	0.069	1	0.001	1	0.082
		10 to 13	2.47 (1.08‐5.61)		3.60 (1.33‐9.76)		1.98 (1.02‐3.83)	
		14+	1.10 (0.54‐2.20)		0.54 (0.15‐2.01)		1.12 (0.59‐2.12)	
SmTSP7	Sex	Female	1	0.798	1	0.117	1	0.186
		Male	1.07 (0.63‐1.81)		2.46 (0.79‐7.56)		2.19 (0.68‐7.00)	
	Age (years)	6 to 9	1	0.309	1	0.224	1	0.969
		10 to 13	1.58 (0.82‐3.06)		0.93 (0.29‐2.96)		1.08 (0.26‐4.58)	
		14+	1.54 (0.82‐2.92)		0.25 (0.05‐1.28)		1.19 (0.30‐4.71)	

OR, odds ratio.

a Adjusted for either sex or age.

### 3.5 Those reinfected after treatment had high pre‐treatment intensity

**Figure 2:**
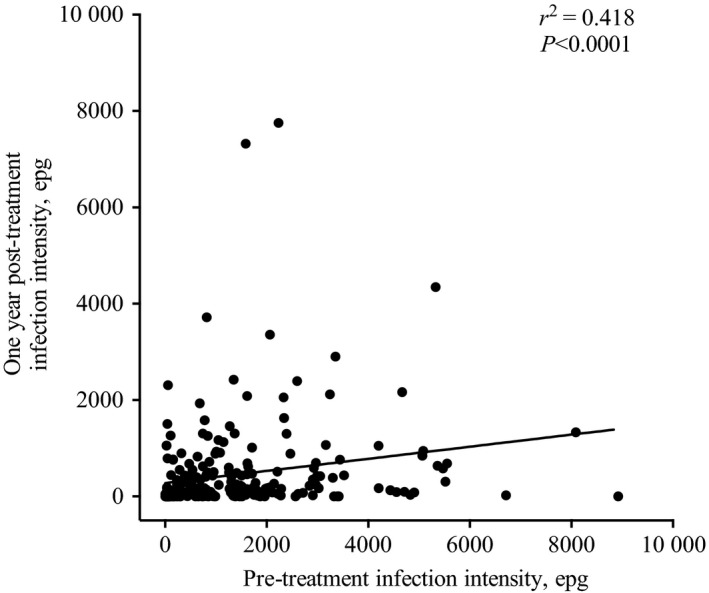
The correlation between the pre‐treatment and one year post‐treatment infection intensity of the study participants (n = 240)

Predisposition describes the phenomenon where those that are reinfected after treatment tend to be the same individuals who had high pre‐treatment intensity. The pre‐ and one year post‐treatment infection intensity in these individuals was positively correlated (*r* ^2 ^= 0.418, *P* < 0.0001) (Figure 2). Having a moderate pre‐treatment intensity decreases the log odds of being resistant to reinfection by 1.4 (*P* = 0.014), compared to having a light infection intensity (Data not shown). In addition, having a heavy pre‐treatment intensity decreased the log odds of being resistant to reinfection by 1.3 (*P* = 0.010), compared to those with a light infection intensity. Those resistant to reinfection were defined as having no eggs one year after treatment. Overall, the effect of pre‐treatment intensity on reinfection intensity (and subsequently resistance to reinfection) was statistically significant after adjusting for age and gender (*P* = 0.011).

### 3.6 Five weeks post‐treatment antibody responses against schistosomula antigens were not associated with one year post‐treatment reinfection intensity among antibody responders

To assess whether antibody responses to schistosomula antigens predicted reinfection, the association between detectable five weeks post‐treatment antibody responses against schistosomula antigens and one year post‐treatment reinfection intensity was investigated. There was no consistent association between the detectable five weeks post‐treatment antibody responses against schistosomula antigens and reinfection intensity one year after PZQ treatment (Table 5).

### 3.7 PZQ treatment differentially affects antibody responses against the recombinant schistosomula antigens among antibody responders

**Figure 3:**
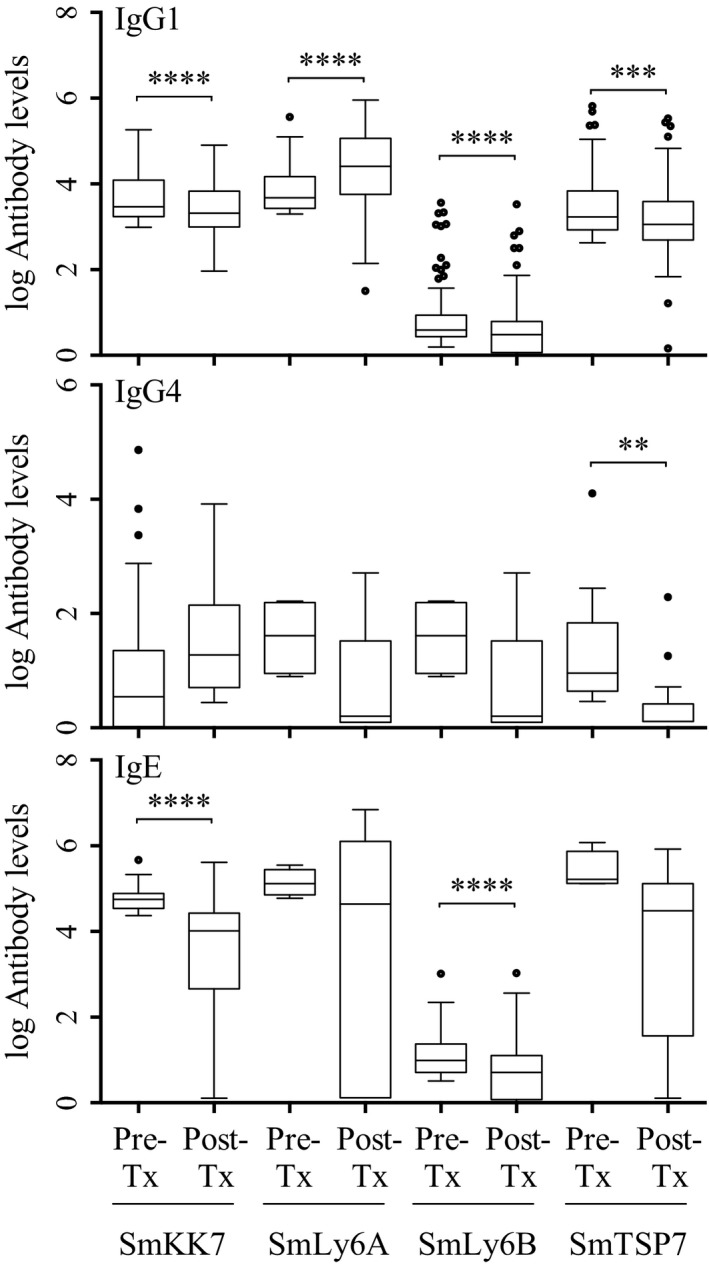
The effect of treatment on antibody responses against the recombinant schistosomula antigens SmKK7 (IgG1: n = 135, IgG4: n = 20 and IgE: n = 51), SmLy6A (IgG1: n = 25, IgG4: n = 3 and IgE: n = 4), SmLy6B (IgG1: n = 163, IgG4: n = 3 and IgE: n = 90) and SmTSP7 (IgG1: n = 90, IgG4: n = 8 and IgE: n = 8) among antibody responders. The boxes indicate the interquartile range with median as the horizontal line, while the whiskers indicate minimum and maximum antibody levels. The dots are outliers. Pre‐Tx, pre‐treatment antibody levels; Post‐Tx, 5 weeks post‐treatment antibody levels; * *P* < 0.0125 (*P*‐value adjusted to take into account the multiple comparison), **P < 0.01, ***P < 0.001, ****P < 0.0001

The antibody levels against the recombinant schistosomula antigens pre‐ and post‐treatment among antibody responders are shown in Figure 3. Among the recombinant antigens, IgG1 to rSmLy6A increased five weeks post‐treatment (*P* < 0.0001). On the other hand, levels of IgG1 to rSmKK7 (*P* < 0.0001), rSmLy6B (*P* < 0.0001) and rSmTSP7 (*P* = 0.0006) and IgE to rSmKK7 (*P* < 0.0001) and rSmLy6B (*P* < 0.0001) dropped five weeks following treatment. IgG4 levels to the schistosomula antigens were not affected by the treatment, with the exception of IgG4 to rSmTSP7 (*P* = 0.0025). The lack of an IgG4 response is likely due to the fact that there were very few IgG4 responders to the recombinant schistosomula antigens.

**Table 5:** Association between detectable five weeks post‐treatment IgG1, IgG4 and IgE responses against schistosomula antigens and reinfection intensity

Antibody	Antigen	Crude OR (95% CI)	*P*‐value	Adjusted^a^ OR (95% CI)	*P*‐value
IgG1	rSmKK7	0.71 (0.34‐1.51)	0.377	1.01 (0.44‐2.30)	0.986
	rSmLy6A	0.27 (0.11‐0.64)	0.004	0.43 (0.16‐1.11)	0.082
	rSmLy6B	0.50 (0.24‐1.07)	0.073	0.80 (0.35‐1.82)	0.592
	rSmTSP7	0.93 (0.43‐1.96)	0.838	1.02 (0.45‐2.31)	0.956
	AWA	0.21 (0.01‐3.58)	0.287	0.62 (0.04‐10.47)	0.741
	SEA	1.59 (0.18‐13.39)	0.668	2.18 (0.23‐19.97)	0.489
IgG4	rSmKK7	0.49 (0.13‐1.73)	0.269	0.58 (0.15‐2.27)	0.439
	rSmLy6A	0.72 (0.19‐2.60)	0.616	0.70 (0.18‐2.76)	0.608
	rSmLy6B	0.37 (0.08‐1.65)	0.193	0.42 (0.09‐2.05)	0.285
	rSmTSP7	1.56 (0.46‐5.16)	0.470	2.17 (0.56‐8.48)	0.265
	AWA	0.89 (0.18‐4.39)	0.885	1.04 (0.19‐5.74)	0.964
	SEA	‐	‐	‐	‐
IgE	rSmKK7	0.74 (0.29‐1.94)	0.546	0.82 (0.29‐2.29)	0.701
	rSmLy6A	0.72 (0.19‐2.59)	0.616	0.54 (0.14‐2.09)	0.371
	rSmLy6B	0.98 (0.45‐2.10)	0.950	1.34 (0.57‐3.12)	0.496
	rSmTSP7	0.68 (0.18‐2.43)	0.551	0.48 (0.12‐1.82)	0.279
	AWA	1.29 (0.59‐2.84)	0.527	1.10 (0.46‐2.62)	0.825
	SEA	1.31 (0.63‐2.78)	0.468	1.19 (0.53‐2.71)	0.671

OR, odds ratio.

a Adjusted for sex and gender.

The effect of PZQ treatment on crude antigens AWA and SEA among antibody responders is shown in Figure S1. IgG1 to the crude antigens AWA (*P* < 0.0001) and SEA (*P* < 0.0001), IgG4 to AWA (*P* < 0.0001) and IgE to AWA (*P* = 0.003) increased five weeks post‐treatment. IgG4 (*P* = 0.526) and IgE (*P* = 0.438) levels to SEA were not affected by treatment.

## 4 DISCUSSION

Schistosomula are vulnerable to both complement and antibody‐dependent cell‐mediated cytotoxicity in in vitro experiments.^9^, ^10^, ^11^, ^41^, ^42^ This makes the newly transformed schistosomulum a plausible source of candidate antigens for a vaccine against *S. mansoni*.^43^ There is information on antibody responses to various *Schistosoma* antigens, but protective antibody responses against schistosomula have not been identified yet and factors that affect these antibody responses are unknown. In this study, we have presented an analysis of antibody responses to schistosomula‐enriched antigens in a cohort from an endemic area. Not surprisingly, we found that being male was associated with high pre‐ and five weeks post‐treatment antibody responses against the schistosomula antigens SmKK7 and rSmLy6a. The sex bias in antibody responses is likely due to the males in the present study spending more time in the lake per day than females, linked to their differing occupations, as has been reported previously.^44^ Although age influences immune responses to *Schistosoma* adult worm antigens,^45^, ^46^ the antibody responses against recombinant schistosomula antigens used in this study were not age dependent. This could imply that age does not influence humoral responses to the early stages of schistosome development. This age‐independent phenomenon may be attributed to the transient nature of the post‐penetration schistosomula in the host skin.^47^ The host is briefly exposed to the schistosomula in a natural situation, and therefore, the required antigen threshold is probably not reached to build immunity.^48^, ^49^ This may suggest that cumulative exposure that is closely related to age of the host may have no impact on immune responses to schistosomula antigens. The implication this has on vaccine design is that vaccines based on schistosomula antigens could provide prolonged exposure to a sufficient amount of schistosomula antigen and generate the same level of protection irrespective of age of the host. On the other hand, the present study observed that sex and age were associated with specific antibody responses to schistosome crude antigens AWA and SEA consistent with previous data.^45^ Where participants had both antibody and cytokine data described in the companion paper (n = 54), very few weak correlations between the antibody and cytokine responses were significant.

Antibody responses to *Schistosoma* antigens are affected by treatment with PZQ.^20^, ^50^ Praziquantel (PZQ) is the most effective drug for treatment of schistosomiasis 51 and is thought to disrupt the regulation of calcium (Ca^2+^) ion permeability through surface membranes.^52^ The resulting Ca^2+^‐induced contractions are sustained, which simultaneously paralyse the parasite and dislodge them from venules. PZQ also damages the worm's body surface by forming fragile blebs and vacuoles in the schistosome tegument.^53^ The blebs and vacuoles rupture, damaging the surface and facilitating host immune responses against schistosomes.^54^, ^55^ In addition, treatment exposes sequestered antigens and antibody responses towards these exposed antigens are elevated.^54^, ^55^ Although the effect of PZQ on the schistosomula is thought to be limited,^56^ treating schistosomula in vitro with drugs (PZQ and oxamniquine) exposes a magnitude of sequestered antigens that are otherwise not accessible on living intact schistosomula.^15^ However, not all *Schistosoma* antigens induce an increase in antibodies following PZQ treatment. For example, IgG1 levels against rSmLy6A and rSmLy6B have been shown to drop following PZQ treatment among antibody responders.^16^ Similarly, we also found a drop in IgG1, IgG4 and IgE to rSmKK7, rSmLy6B and rSmTSP7, respectively, after PZQ treatment among antibody responders. On the other hand, we did observe an increase to SmLy6A. These data are in apparent contradiction to a previously published study showing that antibodies to SmLy6A decreased after treatment.^16^ Similar to the study by Chalmers et al,^16^ the effect of treatment was examined in participants with an antibody response above the cut‐off set using nonendemic European donors at baseline. However, the study sites differed between the two studies, and it is plausible that participants from different endemic areas within a country mount differing antibody responses to the same recombinant antigens. Our findings suggest that there is heterogeneity in the response to *Schistosoma* antigens following treatment and that some antigens induce an immune response and give rise to increased antibodies, while other antigens do not.

As *Schistosoma* has more than one life cycle stage in the definitive host, some *Schistosoma* antigens are shared across these stages of *Schistosoma* including the schistosomulum, the adult and the egg.^57^ As a result, there is potential for cross‐reactive antibody responses to the adult worm, the egg and the schistosomula. The schistosomula antigens tested in the present study SmKK7, SmLy6a, SmLy6b and Smtsp7 are also expressed by the adults.^14^, ^17^, ^57^ These studies have shown that there is a low expression of the mRNA transcripts for these antigens in miracidia, sporocysts and cercariae, increasing in schistosomula and adult worms. Most importantly, the egg that induces the immunopathology associated with chronic schistosomiasis had minimal levels of transcripts. Because treatment with PZQ alters immune responses to adult *Schistosoma* antigens,^58^ it could indirectly modify host immune responses to the schistosomula.^59^ This implies that cross‐reactive antibodies against larvae could be boosted by killing the adults and releasing antigens. Therefore, shared epitopes could be good antigens to study further for vaccines that target both the schistosomula and the adults.

In longitudinal studies, antibody responses to specific schistosome antigens have been shown to correlate with resistance to reinfection.^60^, ^61^ Generally, the antibody responses associated with human resistance to reinfection are directed against the schistosome adult worm and not egg antigens.^62^ This finding may not be surprising as the immune responses to crude adult worm and egg antigens have been widely studied. However, limited work has shown that IgG 32 and IgE 31 antibody responses to larval antigens are associated with resistance to reinfection. By looking at those infected individuals who were cured five weeks following treatment, we related their reinfection intensity to antibody responses to schistosomula antigens pre‐treatment and five weeks post‐treatment. Determinants of reinfection (reviewed in^38^) we considered in this analysis were age and sex. In the present study, pre‐treatment or five weeks post‐treatment antibody responses against schistosomula antigens were not associated with one year post‐treatment reinfection intensity. These findings imply that although the schistosomula antigens tested in the present study were targeted by IgG1 and IgE, the responses were not protective. With the schistosomula as the target of immune attrition, using the radiation‐attenuated cercariae the only experimental vaccine to date to consistently provide high levels of protective immunity (60%‐80%) against challenge,^63^, ^64^, ^65^ there is a need to identify other schistosomula antigens that are targets of protective immunity in humans. It could be that other, perhaps nonprotein, schistosomula molecules such as glycans may be targets of protective immunity in humans. A glycomic analysis of the life cycle of *S. mansoni* shows stage‐specific expression of glycans, including during the schistosomula stage.^66^ Glycans are highly antigenic,^67^ and schistosomula glycans are the dominant targets of antibody responses.^68^, ^69^, ^70^ There is evidence that schistosome glycans induce protective immune responses. For instance, anti‐glycan IgG protects mice against challenge with *S. japonicum*.^71^ Furthermore, glycan‐specific IgG antibodies have been associated with the ability of nonhuman primates to naturally clear *Schistosoma* infections.^72^ In the present study, the recombinant schistosomula proteins were generated in an *E. coli* system without the ability to glycosylate proteins. Therefore, the antibody responses to schistosomula glycoproteins could not be established. As it is now possible to use an engineered *E. coli* system to produce recombinant glycoproteins,^73^, ^74^ schistosomula glycans (in the form of recombinant glycoproteins) should be investigated further for a possible role in human anti‐schistosome immunity and subsequently their potential as vaccine candidates.^75^

In addition to identifying the antibodies that target schistosomula antigens, another important issue to consider during the development of a schistosomiasis vaccine is whether the schistosomula antigens induce IgE. IgE responses to worm antigens correlate with age‐dependent human immunity,^20^, ^76^, ^77^, ^78^ but IgE has also been observed to mediate allergic reactions to helminth antigens in previously infected people.^23^ In the present study, the relatively high IgE responses to rSmKK7 and rSmLy6B would indicate that these antigens could be potentially allergenic if developed further as vaccine candidates and thus are probably not a good avenue for further development. This finding could point to a new class of possible IgE‐binding protein families that have never been seen before. Whether the IgE levels to the schistosomula antigens are sufficient to lead to allergic responses/cross‐reactivity remains to be investigated. Possible vaccine‐induced allergic reactions can be mitigated when selecting vaccine antigens by in silico screening out of potentially allergenic antigens using bioinformatics tools, such as the Structural Database of Allergenic Proteins (SDAP), that predict antigens that IgE binds.^79^ This was, in fact, performed for the schistosomula antigens used in this study, by TheSchistoVac (http://www.theschistovac.eu/). Our present study cohort (the Namoni cohort) is a similar cohort to the Musoli cohort (also in Uganda) but showed that there were individuals with high IgE responses to some of the schistosomula antigens. There appears to be heterogeneity in the response to schistosomula, even between geographically close populations (Musoli vs Namoni). It may be that across a population, there will be people that respond to any novel *Schistosoma* antigen with IgE, and it may not be possible to screen this reactivity out, meaning that there may be some people who will end up with an adverse reaction to any novel antigen. Of course, if the risk is small and can be managed, then the benefit of administering an antischistosome vaccine will far outweigh the risks.

## CONFLICT OF INTEREST

The authors declare that there is no conflict of interest.

## DISCLOSURES

None.

## References

[1] WHO . Schistosomiasis and soil‐transmitted helminthiases: number of people treated in 2015. Wkly Epidemiol Rec. 2016;91(49‐50):585‐595. 27934297

[2] Boisier P , Ramarokoto CE , Ravaoalimalala VE , et al. Reversibility *of Schistosoma mansoni*‐associated morbidity after yearly mass praziquantel therapy: ultrasonographic assessment. Trans R Soc Trop Med Hyg. 1998;92(4):451‐453. 9850407 10.1016/s0035-9203(98)91090-2

[3] Ross AG , Olveda RM , Chy D , et al. Can mass drug administration lead to the sustainable control of schistosomiasis? J Infect Dis. 2015;211(2):283‐289. 25070942 10.1093/infdis/jiu416

[4] Bergquist NR , Leonardo LR , Mitchell GF . Vaccine‐linked chemotherapy: can schistosomiasis control benefit from an integrated approach? Trends Parasitol. 2005;21(3):112‐117. 15734657 10.1016/j.pt.2005.01.001

[5] Wilson RA , Coulson PS . Immune effector mechanisms against schistosomiasis: looking for a chink in the parasite's armour. Trends Parasitol. 2009;25:423‐431. 19717340 10.1016/j.pt.2009.05.011 PMC3686490

[6] McKean JR , Anwar AR , Kay AB . Schistosoma mansoni: complement and antibody damage, mediated by human eosinophils and neutrophils, in killing schistosomula in vitro. Exp Parasitol. 1981;51(3):307‐317. 7227485 10.1016/0014-4894(81)90118-1

[7] Marikovsky M , Levi‐Schaffer F , Arnon R , Fishelson Z . Schistosoma mansoni: killing of transformed schistosomula by the alternative pathway of human complement. Exp Parasitol. 1986;61(1):86‐94. 3943595 10.1016/0014-4894(86)90138-4

[8] Butterworth AE , Sturrock RF , Houba V , Rees PH . Antibody‐dependent cell‐mediated damage to schistosomula in vitro. Nature. 1974;252(5483):503‐505. 4431479 10.1038/252503a0

[9] Khalife J , Dunne DW , Richardson BA , et al. Functional role of human IgG subclasses in eosinophil‐mediated killing of schistosomula of *Schistosoma mansoni* . J Immunol. 1989;142(12):4422‐4427. 2723436

[10] Dunne DW , Richardson BA , Jones FM , Clark M , Thorne KJ , Butterworth AE . The use of mouse/human chimaeric antibodies to investigate the roles of different antibody isotypes, including IgA2, in the killing of *Schistosoma mansoni* schistosomula by eosinophils. Parasite Immunol. 1993;15(3):181‐185. 8316412 10.1111/j.1365-3024.1993.tb00598.x

[11] Butterworth AE , Sturrock RF , Houba V , Mahmoud AA , Sher A , Rees PH . Eosinophils as mediators of antibody‐dependent damage to schistosomula. Nature. 1975;256(5520):727‐729. 1153011 10.1038/256727a0

[12] McLaren DJ , James SL . Ultrastructural studies of the killing of schistosomula of *Schistosoma mansoni* by activated macrophages in vitro. Parasite Immunol. 1985;7(3):315‐331. 3892433 10.1111/j.1365-3024.1985.tb00079.x

[13] Farias LP , Tararam CA , Miyasato PA , et al. Screening the *Schistosoma mansoni* transcriptome for genes differentially expressed in the schistosomulum stage in search for vaccine candidates. Parasitol Res. 2011;108(1):123‐135. 20852890 10.1007/s00436-010-2045-1

[14] Fitzpatrick JM , Peak E , Perally S , et al. Anti‐schistosomal intervention targets identified by lifecycle transcriptomic analyses. PLoS Negl Trop Dis. 2009;3(11):e543. 19885392 10.1371/journal.pntd.0000543 PMC2764848

[15] Reimers N , Homann A , Hoschler B , et al. Drug‐induced exposure of *Schistosoma mansoni* antigens SmCD59a and SmKK7. PLoS Negl Trop Dis. 2015;9(3):e0003593. 25774883 10.1371/journal.pntd.0003593 PMC4361651

[16] Chalmers IW , Fitzsimmons CM , Brown M , et al. Human IgG1 responses to surface localised *Schistosoma mansoni* Ly6 family members drop following praziquantel treatment. PLoS Negl Trop Dis. 2015;9(7):e0003920. 26147973 10.1371/journal.pntd.0003920 PMC4492491

[17] Farias LP , Krautz‐Peterson G , Tararam CA , et al. On the three‐finger protein domain fold and CD59‐like proteins in *Schistosoma mansoni* . PLoS Negl Trop Dis. 2013;7(10):e2482. 24205416 10.1371/journal.pntd.0002482 PMC3812095

[18] Castro‐Borges W , Dowle A , Curwen RS , Thomas‐Oates J , Wilson RA . Enzymatic shaving of the tegument surface of live schistosomes for proteomic analysis: a rational approach to select vaccine candidates. PLoS Negl Trop Dis. 2011;5(3):e993. 21468311 10.1371/journal.pntd.0000993 PMC3066142

[19] van Balkom BW , van Gestel RA , Brouwers JF , et al. Mass spectrometric analysis of the *Schistosoma mansoni* tegumental sub‐proteome. J Proteome Res. 2005;4(3):958‐966. 15952743 10.1021/pr050036w

[20] Dunne DW , Butterworth AE , Fulford AJ , et al. Immunity after treatment of human schistosomiasis: association between IgE antibodies to adult worm antigens and resistance to reinfection. Eur J Immunol. 1992;22(6):1483‐1494. 1601036 10.1002/eji.1830220622

[21] Vereecken K , Naus CW , Polman K , et al. Associations between specific antibody responses and resistance to reinfection in a Senegalese population recently exposed to *Schistosoma mansoni* . Trop Med Int Health. 2007;12(3):431‐444. 17313515 10.1111/j.1365-3156.2006.01805.x

[22] Hotez PJ , Bethony JM , Diemert DJ , Pearson M , Loukas A . Developing vaccines to combat hookworm infection and intestinal schistosomiasis. Nat Rev Microbiol. 2010;8(11):814‐826. 20948553 10.1038/nrmicro2438

[23] Diemert DJ , Pinto AG , Freire J , et al. Generalized urticaria induced by the Na‐ASP‐2 hookworm vaccine: implications for the development of vaccines against helminths. J Allergy Clin Immunol. 2012;130(1):169‐176. e6. 22633322 10.1016/j.jaci.2012.04.027

[24] Bethony JM , Cole RN , Guo X , et al. Vaccines to combat the neglected tropical diseases. Immunol Rev. 2011;239(1):237‐270. 21198676 10.1111/j.1600-065X.2010.00976.x PMC3438653

[25] Rihet P , Demeure CE , Dessein AJ , Bourgois A . Strong serum inhibition of specific IgE correlated to competing IgG4, revealed by a new methodology in subjects from a *S. mansoni* endemic area. Eur J Immunol. 1992;22(8):2063‐2070. 1639104 10.1002/eji.1830220816

[26] Cassard L , Jonsson F , Arnaud S , Daeron M . Fcgamma receptors inhibit mouse and human basophil activation. J Immunol. 2012;189(6):2995‐3006. 22908332 10.4049/jimmunol.1200968

[27] James LK , Till SJ . Potential mechanisms for IgG4 inhibition of immediate hypersensitivity reactions. Curr Allergy Asthma Rep. 2016;16(3):23. 26892721 10.1007/s11882-016-0600-2 PMC4759210

[28] Daeron M , Jaeger S , Du Pasquier L , Vivier E . Immunoreceptor tyrosine‐based inhibition motifs: a quest in the past and future. Immunol Rev. 2008;224:11‐43. 18759918 10.1111/j.1600-065X.2008.00666.x

[29] Butterworth AE , Bensted‐Smith R , Capron A , et al. Immunity in human schistosomiasis mansoni: prevention by blocking antibodies of the expression of immunity in young children. Parasitology. 1987;94(Pt 2):281‐300. 2438629 10.1017/s0031182000053956

[30] Iskander R , Das PK , Aalberse RC . IgG4 antibodies in Egyptian patients with schistosomiasis. Int Arch Allergy Appl Immunol. 1981;66(2):200‐207. 7287200 10.1159/000232819

[31] Rihet P , Demeure CE , Bourgois A , Prata A , Dessein AJ . Evidence for an association between human resistance to *Schistosoma mansoni* and high anti‐larval IgE levels. Eur J Immunol. 1991;21(11):2679‐2686. 1936116 10.1002/eji.1830211106

[32] Dessein AJ , Begley M , Demeure C , et al. Human resistance to *Schistosoma mansoni* is associated with IgG reactivity to a 37‐kDa larval surface antigen. J Immunol. 1988;140(8):2727‐2736. 3128605

[33] McWilliam H , Driguez P , Piedrafita D , McManus D , Meeusen E . Discovery of novel *Schistosoma japonicum* antigens using a targeted protein microarray approach. Parasit Vectors. 2014;7(1):290. 24964958 10.1186/1756-3305-7-290 PMC4080988

[34] Farnell EJ , Tyagi N , Ryan S , et al. Known allergen structures predict *Schistosoma mansoni* IgE‐binding antigens in human infection. Front Immunol. 2015;6:26. 25691884 10.3389/fimmu.2015.00026 PMC4315118

[35] Katz N , Chaves A , Pellegrino J . A simple device for quantitative stool thick‐smear technique in *Schistosomiasis mansoni* . Rev Inst Med Trop Sao Paulo. 1972;14(6):397‐400. 4675644

[36] Montresor A , Crompton DWT , Hall A , Bundy DAP , Savioli L . Guidelines for the evaluation of soil‐transmitted helminthiasis and schistosomiasis at community level. Geneva World Heal Organ. 1998;1‐49.

[37] Tukahebwa EM , Vennervald BJ , Nuwaha F , Kabatereine NB , Magnussen P . Comparative efficacy of one versus two doses of praziquantel on cure rate of *Schistosoma mansoni* infection and re‐infection in Mayuge District, Uganda. Trans R Soc Trop Med Hyg. 2013;107(6):397‐404. 23596262 10.1093/trstmh/trt024

[38] Mbanefo EC , Huy NT , Wadagni AA , Eneanya CI , Nwaorgu O , Hirayama K . Host determinants of reinfection with schistosomes in humans: a systematic review and meta‐analysis. PLoS Negl Trop Dis. 2014;8(9):e3164. 25211227 10.1371/journal.pntd.0003164 PMC4161334

[39] Krogh A , Larsson B , von Heijne G , Sonnhammer EL . Predicting transmembrane protein topology with a hidden Markov model: application to complete genomes. J Mol Biol. 2001;305(3):567‐580. 11152613 10.1006/jmbi.2000.4315

[40] Fitzsimmons CM , Jones FM , Stearn A , et al. The Schistosoma mansoni tegumental‐allergen‐like (TAL) protein family: influence of developmental expression on human IgE responses. PLoS Negl Trop Dis. 2012;6(4):e1593. 22509417 10.1371/journal.pntd.0001593 PMC3317908

[41] McLaren DJ , Ramalho‐Pinto FJ . Eosinophil‐mediated killing of schistosomula of *Schistosoma mansoni* in vitro: synergistic effect of antibody and complement. J Immunol. 1979;123(4):1431‐1438. 479590

[42] Glauert AM , Butterworth AE . Morphological evidence for the ability of eosinophils to damage antibody‐coated schistosomula. Trans R Soc Trop Med Hyg. 1977;71(5):392‐395. 595093 10.1016/0035-9203(77)90036-0

[43] McManus DP , Loukas A . Current Status of vaccines for schistosomiasis. Clin Microbiol Rev. 2008;21(1):225‐242. 18202444 10.1128/CMR.00046-07 PMC2223839

[44] Karanja DM , Hightower AW , Colley DG , et al. Resistance to reinfection with *Schistosoma mansoni* in occupationally exposed adults and effect of HIV‐1 co‐infection on susceptibility to schistosomiasis: a longitudinal study. Lancet. 2002;360(9333):592‐596. 12241930 10.1016/S0140-6736(02)09781-7

[45] Naus CW , Booth M , Jones FM , et al. The relationship between age, sex, egg‐count and specific antibody responses against *Schistosoma mansoni* antigens in a Ugandan fishing community. Trop Med Int Health. 2003;8(6):561‐568. 12791062 10.1046/j.1365-3156.2003.01056.x

[46] de Moira AP , Fulford AJC , Kabatereine NB , Ouma JH , Booth M , Dunne DW . Analysis of complex patterns of human exposure and immunity to *Schistosomiasis mansoni*: the influence of age, sex, ethnicity and IgE. PLoS Negl Trop Dis. 2010;4(9):e820. 20856909 10.1371/journal.pntd.0000820 PMC2939029

[47] He YX , Chen L , Ramaswamy K . *Schistosoma mansoni, S. haematobium*, and *S. japonicum*: early events associated with penetration and migration of schistosomula through human skin. Exp Parasitol. 2002;102(2):99‐108. 12706745 10.1016/s0014-4894(03)00024-9

[48] Woolhouse ME , Hagan P . Seeking the ghost of worms past. Nat Med. 1999;5(11):1225‐1227. 10545976 10.1038/15169

[49] Mutapi F , Burchmore R , Mduluza T , Midzi N , Turner CMR , Maizels RM . Age‐related and infection intensity‐related shifts in antibody recognition of defined protein antigens in a schistosome‐exposed population. J Infect Dis. 2008;198(2):167‐175. 18549316 10.1086/589511

[50] Grogan JL , Kremsner PG , Van Dam GJ , et al. Antischistosome IgG4 and IgE responses are affected differentially by chemotherapy in children versus adults. J Infect Dis. 1996;173(5):1242‐1247. 8627078 10.1093/infdis/173.5.1242

[51] Danso‐Appiah A , Olliaro PL , Donegan S , Sinclair D , Utzinger J . Drugs for treating *Schistosoma mansoni* infection. Cochrane Database Syst Rev. 2013;2(2):CD000528. 10.1002/14651858.CD000528.pub2 PMC6532716 23450530

[52] Doenhoff MJ , Cioli D , Utzinger J . Praziquantel: mechanisms of action, resistance and new derivatives for schistosomiasis. Curr Opin Infect Dis. 2008;21(6):659‐667. 18978535 10.1097/QCO.0b013e328318978f

[53] Mehlhorn H , Kojima S , Rim HJ , et al. Ultrastructural investigations on the effects of praziquantel on human trematodes from Asia: *Clonorchis sinensis*,* Metagonimus yokogawai*,* Opisthorchis viverrini*,* Paragonimus westermani* and *Schistosoma japonicum* . Arzneimittelforschung. 1983;33(1):91‐98. 6338885

[54] Brindley PJ , Strand M , Norden AP , Sher A . Role of host antibody in the chemotherapeutic action of praziquantel against *Schistosoma mansoni*: identification of target antigens. Mol Biochem Parasitol. 1989;34(2):99‐108. 2496307 10.1016/0166-6851(89)90001-7

[55] Brindley PJ , Sher A . The chemotherapeutic effect of praziquantel against *Schistosoma mansoni* is dependent on host antibody response. J Immunol. 1987;139(1):215‐220. 3108397

[56] Silva LM , Menezes RM , de Oliveira SA , Andrade ZA . Chemotherapeutic effects on larval stages of *Schistosoma mansoni* during infection and re‐infection of mice. Rev Soc Bras Med Trop. 2003;36(3):335‐341. 12908033 10.1590/s0037-86822003000300004

[57] Curwen RS , Ashton PD , Sundaralingam S , Wilson RA . Identification of novel proteases and immunomodulators in the secretions of schistosome cercariae that facilitate host entry. Mol Cell Proteomics. 2006;5(5):835‐844. 16469760 10.1074/mcp.M500313-MCP200

[58] Joseph S , Jones FM , Walter K , et al. Increases in human T helper 2 cytokine responses to *Schistosoma mansoni* worm and worm‐tegument antigens are induced by treatment with praziquantel. J Infect Dis. 2004;190(4):835‐842. 15272413 10.1086/422604

[59] Dunne DW , Grabowska AM , Fulford AJC , et al. Human antibody responses to *Schistosoma mansoni*: the influence of epitopes shared between different life‐cycle stages on the response to the schistosomulum. Eur J Immunol. 1988;18(1):123‐131. 2450028 10.1002/eji.1830180119

[60] Jiz M , Friedman JF , Leenstra T , et al. Immunoglobulin E (IgE) responses to paramyosin predict resistance to reinfection with *Schistosoma japonicum* and are attenuated by IgG4. Infect Immun. 2009;77(5):2051‐2058. 19273558 10.1128/IAI.00012-09 PMC2681753

[61] Dunne DW , Butterworth AE , Fulford AJ , Ouma JH , Sturrock RF . Human IgE responses to *Schistosoma mansoni* and resistance to reinfection. Mem Inst Oswaldo Cruz. 1992;87(Suppl 4):99‐103. 10.1590/s0074-02761992000800014 1343933

[62] Odegaard JI , Hsieh MH . Immune responses to *Schistosoma haematobium* infection. Parasite Immunol. 2014;36(9):428‐438. 25201406 10.1111/pim.12084

[63] Eberl M , Langermans JA , Frost PA , et al. Cellular and humoral immune responses and protection against schistosomes induced by a radiation‐attenuated vaccine in chimpanzees. Infect Immun. 2001;69(9):5352‐5362. 11500405 10.1128/IAI.69.9.5352-5362.2001 PMC98645

[64] Yole DS , Reid GD , Wilson RA . Protection against *Schistosoma mansoni* and associated immune responses induced in the vervet monkey Cercopithecus aethiops by the irradiated cercaria vaccine. Am J Trop Med Hyg. 1996;54(3):265‐270. 8600763 10.4269/ajtmh.1996.54.265

[65] Yole DS , Pemberton R , Reid GD , Wilson RA . Protective immunity to *Schistosoma mansoni* induced in the olive baboon *Papio anubis* by the irradiated cercaria vaccine. Parasitology. 1996;1:37‐46. 10.1017/s0031182000065057 8587800

[66] Smit CH , van Diepen A , Nguyen DL , et al. Glycomic analysis of life stages of the human parasite *Schistosoma mansoni* reveals developmental expression profiles of functional and antigenic glycan motifs. Mol Cell Proteomics. 2015;14(7):1750‐1769. 25883177 10.1074/mcp.M115.048280 PMC4587318

[67] Bethony J , Williams JT , Kloos H , et al. Exposure to *Schistosoma mansoni* infection in a rural area in Brazil. II: household risk factors. Trop Med Int Health. 2001;6(2):136‐145. 11251910 10.1046/j.1365-3156.2001.00685.x

[68] Smit CH , Kies CL , McWilliam HEG , Meeusen ENT , Hokke CH , van Diepen A . Local antiglycan antibody responses to skin stage and migratory schistosomula of *Schistosoma japonicum* . Infect Immun. 2015;84(1):21‐33. 26459512 10.1128/IAI.00954-15 PMC4694003

[69] Eberl M , Langermans JA , Vervenne RA , et al. Antibodies to glycans dominate the host response to schistosome larvae and eggs: is their role protective or subversive? J Infect Dis. 2001;183(8):1238‐1247. 11262206 10.1086/319691

[70] Kariuki TM , Farah IO , Wilson RA , Coulson PS . Antibodies elicited by the secretions from schistosome cercariae and eggs are predominantly against glycan epitopes. Parasite Immunol. 2008;30(10):554‐562. 18786069 10.1111/j.1365-3024.2008.01054.x

[71] Gong W , Huang F , Ma Y , et al. Protective immunity against *Schistosoma japonicum* infection can be provided by IgG antibodies towards periodate‐sensitive or periodate‐resistant glycans. Parasit Vectors. 2015;8(1):234‐243. 25907161 10.1186/s13071-015-0842-1 PMC4408597

[72] Yang YYM , Li XH , Brzezicka K , et al. Specific anti‐glycan antibodies are sustained during and after parasite clearance in *Schistosoma japonicum*‐infected rhesus macaques. PLoS Negl Trop Dis. 2017;11(2):e0005339. 28151933 10.1371/journal.pntd.0005339 PMC5308859

[73] Valderrama‐Rincon JD , Fisher AC , Merritt JH , et al. An engineered eukaryotic protein glycosylation pathway in *Escherichia coli* . Nat Chem Biol. 2012;8(5):434. 22446837 10.1038/nchembio.921 PMC3449280

[74] Breyer CA , de Oliveira MA , Pessoa A . Expression of glycosylated proteins in bacterial system and purification by affinity chromatography In: Picanço‐Castro V., Swiech K. (eds) Recombinant Glycoprotein Production. Methods in Molecular Biology, New York, NY: Humana Press; 1674. 10.1007/978-1-4939-7312-5_14 28921437

[75] Nyame AK , Lewis FA , Doughty BL , Correa‐Oliveira R , Cummings RD . Immunity to schistosomiasis: Glycans are potential antigenic targets for immune intervention. Exp Parasitol. 2003;104(1‐2):1‐13. 12932753 10.1016/s0014-4894(03)00110-3

[76] Webster M , Fallon PG , Fulford AJ , et al. IgG4 and IgE responses to *Schistosoma mansoni* adult worms after treatment. J Infect Dis. 1997;175(2):493‐494. 9203682 10.1093/infdis/175.2.493

[77] Fitzsimmons CM , Jones FM , Pinot de Moira A , et al. Progressive cross‐reactivity in IgE responses: an explanation for the slow development of human immunity to schistosomiasis? Infect Immun. 2012;80(12):4264‐4270. 23006852 10.1128/IAI.00641-12 PMC3497412

[78] Zhang Z , Wu H , Chen S , et al. Association between IgE antibody against soluble egg antigen and resistance to reinfection with *Schistosoma japonicum* . Trans R Soc Trop Med Hyg. 1997;91(5):606‐608. 9463682 10.1016/s0035-9203(97)90047-x

[79] Ivanciuc O , Schein CH , Braun W . SDAP: database and computational tools for allergenic proteins. Nucleic Acids Res. 2003;31(1):359‐362. 12520022 10.1093/nar/gkg010 PMC165457

