# Supplementary material for: Antibody responses to Schistosoma mansoni schistosomula antigens
Source: Parasite Immunol. 2018 Oct 14;40(12):e12591. doi: 10.1111/pim.12591 (PMC6492298; doi:10.1111/pim.12591)
Supplement: Supplementary file 1 [file PIM-40-na-s001.doc]

**Figure S1** The effect of treatment on the antibody responses against the *S. mansoni* crude antigens AWA (IgG1; n=201, IgG4; n=200 and IgE; n=109) and SEA (IgG1; n=203, IgG4; n=211 and IgE; n=67) among antibody responders. The boxes indicate the interquartile range with median as the horizontal line while the whiskers indicate minimum and maximum antibody levels. The dots are outliers. Pre-Tx, Pretreatment antibody levels; Post-Tx, 5-week post-treatment antibody levels; *p<0.025 (p-value adjusted to take into account the multiple comparison), **p<0.01, ***p<0.001, ****p<0.0001
