# Supplementary material for: Antibody responses to Schistosoma mansoni schistosomula antigens
Source: Parasite Immunol. 2018 Oct 14;40(12):e12591. doi: 10.1111/pim.12591 (PMC6492298; doi:10.1111/pim.12591)
Supplement: Supplementary file 2 [file PIM-40-na-s002.docx]

**Supplementary Tables**

**Table S1.** Water Contact Details of the Study Participants (N=226)

| Water contact activity | **Level** | **n (%)** |
| --- | --- | --- |
| Spending time in lake (hours/day) | <0.5 | 83 (36.7) |
|  | 0.5-1 | 108 (47.8) |
|  | 1-2 | 13 (5.7) |
|  | 2-3 | 6 (2.7) |
|  | 3-4 | 5 (2.2) |
|  | >4 | 11 (4.9) |
| Fishing in the lake | No | 201 (88.9) |
|  | Yes | 25 (11.1) |
| Washing or processing fish in lake | No | 188 (83.2) |
|  | Yes | 38 (16.8) |
| Washing clothes or utensils in lake | Yes | 176 (77.9) |
|  | No | 50 (22.1) |
| Swimming / bathing / playing in the lake | No | 164 (72.6) |
|  | Yes | 62 (27.4) |
| Fetching water from the lake | No | 206 (91.2) |
|  | Yes | 20 (8.8) |
| Going to the lake for transport | No | 174 (77.0) |
|  | Yes | 52 (23.0) |
| Going to the lake for farm irrigation | No | 183 (81.0) |
|  | Yes | 43 (19.0) |
| Going to the lake for anything else | No | 119 (52.7) |
|  | Yes | 107 (47.3) |

**Table S2.** Factors Associated with Five-Week Post-Treatment IgG1, IgG4 and IgE Levels Against Crude Antigens

| Antigen | Factor | Level | IgG1 | | IgG4 | | IgE | |
| --- | --- | --- | --- | --- | --- | --- | --- | --- |
|  |  |  | Adjusted† OR‡  (95% CI) | P–value | Adjusted† OR‡  (95% CI) | P–value | Adjusted† OR‡  (95% CI) | P–value |
| AWA | Sex | Female | 1 |  | 1 | 0.631 | 1 | 0.015 |
|  |  | Male | - | - | 1.33 (0.42–4.27) |  | 2.01 (1.15–3.55) |  |
|  | Age | 6 to 9 | 1 | 0.625 | 1 | 0.722 | 1 | 0.025 |
|  | (years) | 10 to 13 | 4.09 (0.78–21.38) |  | 3.32 (0.09–13.23) |  | 2.19 (1.12–4.28) |  |
|  |  | 14+ | 0.73 (0.25–2.10) |  | 1.11 (0.41–3.05) |  | 2.17 (1.13–4.15) |  |
| SEA | Sex | Female | 1 | 0.372 | 1 | 0.754 | 1 | 0.128 |
|  |  | Male | 1.54 (0.60–1.71) |  | 0.73 (0.10–5.38) |  | 1.50 (0.89–2.53) |  |
|  | Age | 6 to 9 | 1 | 0.185 | 1 | 0.861 | 1 | 0.379 |
|  | (years) | 10 to 13 | 468 (0.51–43.20) |  | 3.54 (0.35–35.33) |  | 0.82 (0.41–1.65) |  |
|  |  | 14+ | 0.41 (0.13–1.35) |  | 1.41 (0.27–1.7.37) |  | 0.89 (0.45–1.74) |  |
| † OR Odds Ratio, ‡ Adjusted for either sex age | | | | | | | | |

**Table S3.** Factors Associated with Five-Week Post-Treatment IgG1, IgG4 and IgE Levels Against Schistosomula Antigens

| Antigen | Factor | Level | IgG1 | | IgG4 | | IgE | |
| --- | --- | --- | --- | --- | --- | --- | --- | --- |
|  |  |  | Adjusted† OR‡  (95% CI) | P–value | Adjusted† OR‡  (95% CI) | P–value | Adjusted† OR‡  (95% CI) | P–value |
| SmKK7 | Sex | Female | 1 | 0.125 | 1 | 0.001 | 1 | 0.308 |
|  |  | Male | 1.52 (0.89–2.59) |  | 3.49 (1.63–7.44) |  | 1.37 (0.74–2.50) |  |
|  | Age  (years) | 6 to 9 | 1 | 0.070 | 1 | 0.254 | 1 | 0.528 |
|  |  | 10 to 13 | 1.48 (0.75–2.92) |  | 1.96 (0.91–4.18) |  | 0.88 (0.42–1.84) |  |
|  |  | 14+ | 0.70 (0.37–1.32) |  | 0.66 (0.29–1.53) |  | 0.96 (0.47–1.93) |  |
| SmLy6a | Sex | Female | 1 | 0.038 | 1 | 0.120 | 1 | 0.146 |
|  |  | Male | 1.76 (1.03–3.02) |  | 1.90 (0.85–4.25) |  | 1.84 (0.81–4.18) |  |
|  | Age | 6 to 9 | 1 | 0.002 | 1 | 0.822 | 1 | 0.391 |
|  | (years) | 10 to 13 | 1.51 (0.78–2.90) |  | 1.38 (0.51–3.74) |  | 0.51 (0.09–2.86) |  |
|  |  | 14+ | 0.47 (0.24–0.90) |  | 1.16 (0.43–3.16) |  | 0.65 (0.14–3.08) |  |
| SmLy6b | Sex | Female | 1 | 0.718 | 1 | 0.602 | 1 | 0.421 |
|  |  | Male | 1.11 (0.64–1.90) |  | 1.22 (0.57–2.62) |  | 1.24 (0.72–2.13) |  |
|  | Age | 6 to 9 | 1 | 0.0062 | 1 | 0.135 | 1 | 0.149 |
|  | (years) | 10 to 13 | 2.47 (1.08–5.61) |  | 3.60 (1.33–9.76) |  | 1.98 (1.03–3.83) |  |
|  |  | 14+ | 1.10 (0.54–2.20) |  | 0.54 (0.15–2.01) |  | 1.12 (0.59–2.12) |  |
| SmTSP7 | Sex | Female | 1 | 0.965 | 1 | 0.045 | 1 | 0.168 |
|  |  | Male | 1.01 (0.60–1.71) |  | 2.66 (0.85–4.25) |  | 1.79 (0.78–4.16) |  |
|  | Age | 6 to 9 | 1 | 0.188 | 1 | 0.633 | 1 | 0.131 |
|  | (years) | 10 to 13 | 1.58 (0.82–3.06) |  | 0.93 (0.29–2.96) |  | 1.09 (0.26–4.58) |  |
|  |  | 14+ | 1.54 (0.82–2.92) |  | 0.25 (0.05–1.28) |  | 1.19 (0.30–4.71) |  |
| † OR Odds Ratio, ‡ Adjusted for either sex age | | | | | | | | |
